# An expanded phylogeny of social amoebas (Dictyostelia) shows increasing diversity and new morphological patterns

**DOI:** 10.1186/1471-2148-11-84

**Published:** 2011-03-31

**Authors:** Maria Romeralo, James C Cavender, John C Landolt, Steven L Stephenson, Sandra L Baldauf

**Affiliations:** 1 Department of Systematic Biology, Evolutionary Biology Centre, Norbyvägen 18D, University of Uppsala, SE-75236 Uppsala, Sweden; 2 Department of Environmental and Plant Biology, Ohio University, Athens, OH 45701, USA; 3 Department of Biology, Shepherd University, Shepherdstown, WV 25443, USA; 4 Department of Biological Sciences, University of Arkansas, Fayetteville, AR 72701, USA

## Abstract

**Background:**

Social Amoebae or Dictyostelia are eukaryotic microbes with a unique life cycle consisting of both uni- and multicellular stages. They have long fascinated molecular, developmental and evolutionary biologists, and *Dictyostelium discoideum *is now one of the most widely studied eukaryotic microbial models. The first molecular phylogeny of Dictyostelia included most of the species known at the time and suggested an extremely deep taxon with a molecular depth roughly equivalent to Metazoa. The group was also shown to consist of four major clades, none of which correspond to traditional genera. Potential morphological justification was identified for three of the four major groups, on the basis of which tentative names were assigned.

**Results:**

Over the past four years, the Mycetozoan Global Biodiversity Survey has identified many new isolates that appear to be new species of Dictyostelia, along with numerous isolates of previously described species. We have determined 18S ribosomal RNA gene sequences for all of these new isolates. Phylogenetic analyses of these data show at least 50 new species, and these arise from throughout the dictyostelid tree breaking up many previously isolated long branches. The resulting tree now shows eight well-supported major groups instead of the original four. The new species also expand the known morphological diversity of the previously established four major groups, violating nearly all previously suggested deep morphological patterns.

**Conclusions:**

A greatly expanded phylogeny of Dictyostelia now shows even greater morphological plasticity at deep taxonomic levels. In fact, there now seem to be no obvious deep evolutionary trends across the group. However at a finer level, patterns in morphological character evolution are beginning to emerge. These results also suggest that there is a far greater diversity of Dictyostelia yet to be discovered, including novel morphologies.

## Background

The Dictyostelia (social amoebae) are common soil dwelling amoeba most often isolated from the leaf litter decomposition zone of forest soils [1]. The first known dictyostelid was isolated by Brefeld [2], but by 1940 still only ten species were recognized. Now, largely due to the work of a handful of people, this number exceeds 100 [3]. Dictyostelia are perhaps best known for the model organism *Dictyostelium discoideum*, and the fact that their home clade, the Amoebozoa is the sister group to the Opisthokonts (holozoa + holofungi) [4].

The dictyostelids possess an unusual life cycle, known almost exclusively from laboratory observations [1]. Throughout most of the asexual cycle individual dictyostelid amoebae feed upon bacteria and multiply by binary fission. When food becomes scarce, amoebae aggregate by the tens of thousands gradually forming a multicellular entity with differentiated cell types. The aggregate then develops into a slug or pseudoplasmodium, a true multicellular polarized unit. The slug moves as a more or less coherent unit, with a head region seeking environmental conditions suitable for the formation of fruiting bodies [5]. Fruiting bodies vary widely in morphology but essentially consist of one or more stalks, in most cases composed of dead cells supporting one or more distinct spore masses (sori) in a variety of arrangements [1]. The entire process is coordinated by the production of chemo-attractants, a process that is well characterized in the model organism *D. discoideum *but mostly unknown in other species [3,5,6]. Thus, in the dictyostelid life cycle growth and development are completely separate; growth occurs only in the unicellular amoebae, and once aggregation starts, cell division ceases and differentiation and morphogenesis can begin [5,7].

Less is known about the sexual cycle of dictyostelids, which culminates in the formation of a macrocyst. This cycle can be between both homothallic (self-fertile) and heterothallic forms [1]. A recent study identified the mating-type locus for the model species *Dictyostelium discoideum *[8]. As with fruiting bodies, macrocyst formation begins with an aggregative process. However, instead of forming a slug, two of the aggregating amoebae fuse, consume the remaining cells and then encyst. Macrocysts were only recognized as the sexual stage of Dictyostelia in the l960s [9,10], and although they were probably present in the last common ancestor of the taxon [11], there are many species for which this stage has yet to be observed. Macrocysts also serve as a resistant stage for surviving sub-optimal growth conditions [3]. Thus, dictyostelids have three ways of dealing with unfavourable conditions: (a) encystment of individual amoebae (microcysts), (b) formation of sexual macrocysts and (c) formation of multicellular fruiting bodies containing spores.

Traditionally, classification of dictyostelids has been based on morphology [1,12]. The characters used range from aspects of the initial aggregation stage including the overall pattern (mound, radiate) and type of aggregative signalling molecule (acrasin: cAMP, glorin, folate, etc), to structural features of the final fruiting body. The latter include features such as the type of growth (clustered, gregarious, coremiform or solitary) and branching pattern, characteristics of the spores such as their overall shape (round or elliptical) and the presence or absence of polar granules inside them, etc [1,12]. Based on these characters, the three traditional genera of Dictyostelia were defined--*Acytostelium *[13] with acellular stalks, *Dictyostelium *[2] with cellular stalks and mostly unbranched or sparsely branched fruiting bodies and *Polysphondylium *[1] with regularly spaced whorls of lateral branches on cellular stalks.

However, a cladistic study of dictyostelia morphology first suggested [14] and molecular phylogeny later confirmed [11,15] that this traditional morphology based taxonomy is deeply flawed. Instead, molecular phylogeny grouped nearly all of the species known at the time into four major clades. None of these major clades correspond to traditional genera indicating that there is far less pattern in dictyostelia morphological evolution at the deepest taxonomic levels than initially thought [11]. SSU rDNA analyses also depicted a very deep phylogeny with many large gaps (long unbroken branches), suggesting large numbers of missing species and/or highly uneven SSU rDNA evolutionary rates [11]. Meanwhile finer level studies including multiple isolates of several "species" showed that these are not always molecularly similar. Such morphologically similar but phylogenetically distinct taxa are referred to individually as cryptic species [16,17] and together as species complexes. Species complexes are scattered throughout the dictyostelid tree, and are found in all four major groups [16,17]. The combination of a deep taxon littered with cryptic species suggests that much dictyostelid diversity remains to be described. Filling in these gaps should help us to develop a better understanding of evolutionary trends across the group.

During the period of 2000 to 2009, the number of described species of Dictyostelia essentially doubled. This was primarily the result of the "Global Biodiversity Survey of Eumycetozoans" project (PBI), mandated to investigate the diversity of dictyostelids and other eumycetozoans throughout the world. Soil samples were collected at various localities, emphasizing (a) regions of the Southern Hemisphere where there were few or no previous records and (b) areas of the Northern Hemisphere that have received relatively little study. Together these include Australia (the mainland and Tasmania), New Zealand, South Africa, Patagonia, northern Thailand, Laos and East Africa. Additional samples were obtained from localities in Central America, Alaska and several islands (Ascension Island, Cocos Island, Puerto Rico and Madagascar). The range of vegetation types sampled included grassland, savanna, shrubland, southern beech forest, *Eucalyptus *forest, lowland tropical rain forest, montane tropical forest and tropical monsoon forest.

We have determined complete SSU rDNA sequences from all new species isolated from PBI project samples, as well as additional isolates of previously described species. The new species and isolates are dispersed across the tree, filling in many gaps and indicating that there are eight major divisions of Dictyostelia rather than the previously recognized four. Most importantly, the new species expand the known morphological diversity of all four previously recognized major clades, indicating that morphology in Dictyostelia is even more plastic than previously realized. At the same time, some patterns at higher taxonomic levels are beginning to emerge.

## Results

Fifty-four new isolates of dictyostelia were identified and subjected to morphological and molecular characterization. New isolates were collected from New Zealand [18], Australia [19], Argentina [20], Laos, northern Thailand, different localities in the USA (including Alaska), Europe (Portugal and Sweden), Africa and the Caribbean. In all, more than 500 samples were collected in more than 10 countries on 5 continents (see Additional file 1). All isolates were identified from laboratory incubated soil samples using traditional culture techniques [1] and re-cultured from single sporophores before DNA extraction.

Complete nuclear small subunit ribosomal RNA gene (SSU rDNA) sequences were determined for all new isolates and added to an existing alignment [11] including all available unique dictyostelia SSU rDNA sequences. A conservative core of 1676 universal and 324 group-specific alignment positions were used to construct phylogenetic trees. The remaining more rapidly evolving segments of SSU rDNA that are often used to delineate species in other taxa, are unalignable across the Dictyostelia and often difficult to align even within major groups [17].

**Figure 1:**
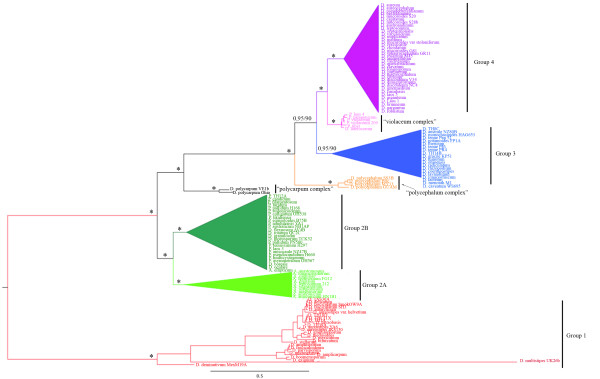
**Phylogeny of the Dictyostelia based on SSU rDNA sequences**. The tree shown is the optimal topology obtained by both Bayesian inference and maximum likelihood searches. The tree identifies eight major taxonomic divisions, which are indicated by separate colors. The tree includes all known described and undescribed species of *Dictyostelium *(D), *Polysphondylium *(P) and *Acytostelium *(A). Branch lengths are the average of all trees recovered during the BI search after discarding a burn-in of 20% and are drawn to scale as indicated by the scale bar at the lower left. Support from BI posterior probabilities and 100 ML bootstrap replicates are respectively indicated to the left and right of slashes (/) on the relevant branches for values above 0.7 biPP and 50% mlBP. Values of 100% mlBP and 1.00 biPP are indicated as *. The tree is rooted according to [11].

**Figure 2:**
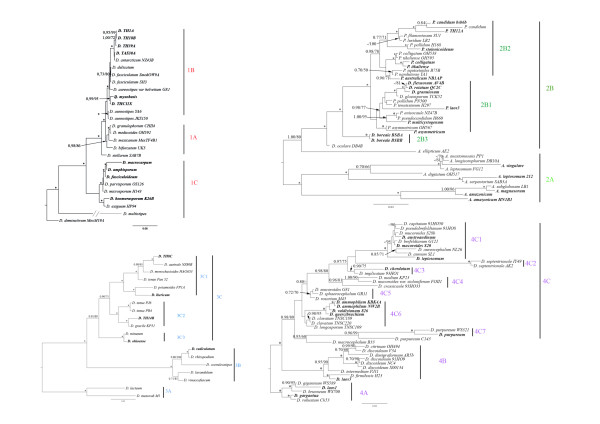
**Detailed Subtrees for Dictyostelid Major Groups 1-4**. The large 18S rDNA subtrees from the Figure 1 are shown in detail for dictyostelid major groups 1-4 (parts A-D). The names in bold correspond to the new species sequenced for this study. Major clades are indicated to the right of the figures with brackets and numerical clade designations as defined in [17]. For Group 1 (A) note that the branch to *D. multistipes *has been reduced in length for this figure. The actual length of the branch can be seen in Figure 1. For B) note that the species listed originally as *P. ****nandutensis ***was finally published as ***P. arachnoideum***.

Phylogenetic analysis of dictyostelia SSU rDNA sequences produces a single well-resolved tree (Figure 1), with strong support for nearly all branches (Figure 2). The strong resolution even at fine levels appears to be due to new species filling out the tree, obviating the need to use more rapidly evolving markers such as the internal transcribed spacer (ITS) to resolve terminal clades [17]. The new phylogeny continues to show the four previously identified major groups with strong support [11]. In addition, three previously isolated and inconsistently resolved branches [11] are now seen to form major divisions in their own right (Figure 1). We refer to these new groups here as the "polycarpum", "polycephalum" and "violaceum" complexes in order to retain the original group numbering scheme [11] until formal names can be assigned. In addition the new species further emphasize the deep split in Group 2, which is now very strongly supported (80-100% mlBP, 1.0 biPP, Figure 1). Therefore, we now recognize these as two separate major groups, Group 2A and Group 2B (Figure 1, Figure 2B).

Thus, in total we now recognize eight major divisions of Dictyostelia, none of which correspond to traditional genera. The closest to a traditional genus is Group 2A, which consists entirely of most of the known acytostelids. However, a single acytostelid, *A. ellipticum*, is found in Group 2B, thus making the traditional *Actyostelium *paraphyletic (Figure 2B). Similarly, Group 2B includes most of the known polysphondylids, but some polysphondylids, including the original "type" species *P. violaceum*, are found a substantial distance away in the "violaceum" complex, which appears as the sister lineage to Group 4 (Figure 1). Thus, the traditional *Polysphondylium *is both paraphyletic and polyphyletic.

Most importantly, the new isolates also substantially expand the morphological diversity of the original four major groups. These groups are described below following the terminology of Raper [1].

### Group I

Group I includes ten new isolates, all of which exhibit the traditional general dictyostelid morphology (Figure 2). That is, they have cellular stalks and lack regular whorls of side (lateral) branches. Nonetheless, these species vary widely in sporophore size and morphology, ranging from solitary to clustered sorocarps, with or without irregular lateral branches, which, when present vary in form (Additional file 1). All of the new isolates can be recognized as new species on the basis of their morphology (Additional file 1), and this is supported in all cases by SSU rDNA phylogeny (Figure 2).

Many of the new isolates cluster with species that have been previously isolated but from very different localities. For example, the only new Group 1 species from Tasmania (TAS30A) clusters with *Dictyostelium antarcticum *as a sister group to three new species from Thailand (subclade 1B, Figure 2A). Four new species from Argentina (*D. macrocarpum*, *D. fasciculoideum*) cluster together with new species from the USA (*D. amphisporum*) and Australia (*D. boomerasporum*), nearly doubling the size of subclade 1C (Figure 2A).

A number of the new Group 1 species fall along previously unbroken long branches (Figure 2) supporting the idea that we may be able to fill in more of the remaining large gaps in this part of the tree with extant missing species. The fact that the new Group 1 species tend to have small delicate sorocarps (e.g., *D. deminutivum*, *D. stellatum*) and may be more abundant in the nutrient-poor habitats that are especially poorly sampled [21], further suggests that broader sampling could potentially yield considerably more new Group 1 species.

### Group 2

Group 2 as a whole includes 19 new isolates, of which at least 12 appear to be new species (Figure 2B). The group remains the most morphologically diverse, including examples of all three traditional genera (Figure 2B). Nonetheless, subclade 2A by itself is extremely homogeneous consisting of all but one of the acytostelids. Subclade 2B, on the other hand, is extremely heterogeneous including one acytostelid (*Acytostelium ellipticum*), all small non-pigmented polysphondylids and six dictyostelids. This makes these two subclades highly distinct from each other, and, with the additional new isolates, their separation is strongly supported by phylogenetic analysis (1.00 biPP, 100% mlBP, Figure 1). Therefore, we suggest that they should be treated as two separate major groups.

Four of the subclade 2B dictyostelids are new, including a new deep branch, *D. boreale*, while the other previously solo deep branch, *D. gloeosporum *(from Japan), is now joined by three new species from Australia (Additional file 1). This makes three separate dictyostelid clades branching with or among the pale polysphondylids. Since the *D. boreale *and *D. oculare *sequences form separate sister lineages to the pale polysphondylids, this suggests that the latter evolved from a dictyostelid-type ancestor. Meanwhile, since a clade of dictyostelids is nested within a clade of pale polysphondylids (subclade 2B1, 1.00 biPP, 95% mlBP, Figure 2B), this indicates that there has been at least one reversion from the polysphondylid to dictyostelid type. Among the new polysphondylids, *P. stolonicoideum *is particularly interesting in having a strong tendency to become decumbent, prostrate and then stoloniferous [19]. Thus, the initial sporophore falls over, and when the spore head contacts the substrate, the spores germinate and immediately re-initiate sporophore formation. This ability of spores to differentiate into stalk and spore cells immediately after germination is also seen in the Group 4 species *D. mucoroides variety stoloniferum *[22] and, less often, *D. implicatum*.

Subclade 2A includes three new species of Acytostelids and additional isolates of *A. leptosomum *and *A. amazonicum *(Figure 2B, Additional file 1). Neither new isolate group with the corresponding previously sequenced isolate, which is the first indication of cryptic species in this group. It is not too surprising that there should be cryptic species here as these species are tiny and delicate with few morphological characters to distinguish them. These are also among the slowest growing species of Dictyostelia, they tend to be restricted in distribution, and many are rare to very rare. Thus, while some of the gaps in subclade 2A may be due to extinction, it is also very likely that there is major under-sampling of what appears from the phylogeny to be a very deep lineage within Dictyostelia (Figure 1).

### Group 3

Group 3 consists of twenty dictyostelium isolates, including five new species (Figure 2C, Additional file 1). With the addition of these new species, the group is now completely resolved phylogenetically with good support for all branches. The new species are from Thailand (TH8C and TH14B), southern Portugal (*D. ibericum*), the USA (*D. ohioense*), and Australia (*D. radiculatum*) (Additional file 1). *Dictyostelium radiculatum *has a sporophore with a crampon base (Additional file 1) and groups with all the other crampon-based species, forming the molecularly and morphologically very distinct subclade 3B (1.00 biPP, 100% mlBP, Figure 2C).

### Group 4

Group 4 continues to be the most species-rich group, including 12 new isolates, of which nine appear to be new species (Additional file 1, Figure 2D). Some of the new species such as *D. valdivianum, D. austroandinum *and *D. ammophilum *have clustered and coremiform sorocarps, presence of branches and polar granules inside their spores, changing previous morphological patterns for the group. The new species *D. chordatum *groups together with *D. implicatum *(100% mlBP, 1.0 biPP, Figure 2D), both of which frequently form tangled sorocarps resulting in a rope-like appearance. This tangling indicates the loss of proximity inhibition, normally the result of NH_4 _sensing [21]. The new Group 4 species, *D. gargantuum*, is the single largest dictyostelid described thus far, with huge (by microbial standards) solitary sorocarps (3-8 mm or more tall if erect and 20-30+ mm or more if prone; Additional file 1) [23].

### Additional major lineages

The previous global SSU rDNA phylogeny of Dictyostelia also showed three lineages without clear affinity to any major group - *D. polycarpum *(2 isolates), *D. polycephalum *(one isolate) and *D. laterosorum *+ *P. violaceum *(2 isolates) [11]. Since these "lineages" consisted of only one or two sequences in the original phylogeny, it was not clear if they represent additional major groups or simply a few unusual SSU rDNA sequences [11]. With the new isolates there are now three additional sequences in the polycephalum lineage and four in the violaceum lineage, with the result that all three lineages now show considerable molecular depth and are strongly supported as distinct (Figure 1). This indicates that they correspond to major groups in their own right, provisionally referred to here as the "polycarpum", "polycephalum" and "violaceum" complexes (Figure 1).

All three complexes have strong morphological identities and large sequence depths. The polycarpum "isolates" both form small delicate clustered sporophores, but their SSU rDNA sequences are as different from each other as nearly any two species in Group 4 [11]. A similar level of sequence difference is seen among the four isolates of polycarpum, all of which form relatively large robust sporophores with a single stalk surmounted by a small cluster of spore heads (Additional file 1). The violaceum complex, on the other hand, shows a similar level of sequence divergence but five very distinct morphologies (Additional file 1). It should be noted that although this violaceum complex appears as the sister group to Group 4 in the SSU rDNA tree, it is clearly distinct from it and the SSU ITS sequences of the two groups are unalignable [17].

## Discussion

Largely due to the efforts of the Global Survey of Eumycetozoa, the number of known species in Dictyostelia has nearly doubled since 2006, and SSU rDNA phylogeny now shows eight major divisions. Three of these correspond to lineages that consisted of only one or two sequences in the original phylogeny (11), and two groups arise from a strongly supported deep split in Group 2 (Figure 2B). Many of the long branches in the initial SSU rDNA phylogeny [11] are also now broken with new species and/or additional isolates of previously described species. Taken together, this confirms previous suggestions that the diversity of Dictyostelia is seriously under-sampled and thus many of the remaining long unbroken branches may also represent extant missing taxa. This is consistent with the still small number of described species and the apparent antiquity of the taxon, estimated at 400 myr for Group 4 alone [24].

### Hidden diversity

This missing diversity of Dictyostelia seems to result from a combination of factors. There is a high level of cryptic diversity; of the 17 "species" for which multiple isolates are examined here only eight are monophyletic (Figure 1, Figure 2) [17]. Even when isolates of the same species group together, they can show tremendous sequence difference, for example the isolates of *P. candidum *and *P. asymetricum *in Group 2B (Figure 2B), of *D. purpureum *in Group 4 (Figure 2D) [16] and of course the isolates of *D. polycephalum *and of *D. polycarpum *(Figure 1). Another factor is the heterogeneous nature of the soil substrate, which is nearly impossible to sample exhaustively [25,26]. Some of the missing diversity may also be due to a previously unrecognized ecological diversity. Forest soils have long been considered the primary habitat of Dictyostelia [1]. However, many of the newly discovered species were found in nutrient poor habitats, for example higher forest elevations, bogs, etc., where there has been little previous sampling. Thus there are whole classes of substrates that have not been examined at all for these species.

### Morphological traits

Molecular phylogeny has disproved long-standing theories for deep evolutionary trends in Dictyostelia, particularly the evolution from simple (acytostelid) to relatively complex (polysphondylid) morphologies [11]. The high level of cryptic species also shows that morphological characters are often unreliable for distinguishing species (16, 17, Figure 2). Nonetheless, all the newly proposed species were first identified based on morphology and roughly half (8 out of 17) of the species for which multiple isolates have been examined form monophyletic groups (Figure 1, Figure 2). Thus, patterns at deeper taxonomic level may also exist. A mapping of morphological characters onto the initial dictyostelid phylogeny suggested some deep evolution trends for the four major groups [11,27]. Although the new species invalidate some of these, particularly for Groups 1 and 4, some patterns are now suggested at finer taxonomic levels.

### Group I

The 2006 analysis identified small spores as the main morphological character for Group 1 [11]. However, the newly described and distantly related Group 1 species *D. boomeransporum *and *D. myxobasis *have some of the largest spores yet seen in the Dictyostelia (Additional file 1). On the other hand, it appears that nearly all species in Group 1 have sporophores with irregular branches. The exceptions to this are some of the isolates from Thailand, which tend to be generally unbranched. However, these species form a single tight clade nested well within Group 1 (Figure 2), so only a single loss is required to explain the distribution of this character in Group 1. All Group 1 species examined thus far also have consolidated polar granules inside their spores.

### Groups 2A and 2B

Group 2 showed a deep split in the original SSU rDNA phylogeny, but this received only moderate support (0.89 biPP, 66% mlBP, [11]). With the addition of new species, the split is now well supported (1.0 biPP, 80% mlBP, Figure 2), defining two very distinct major divisions. Group 2A is very homogeneous, consisting of all examined acytostelids except *A. ellipticum*. These species have the distinct combined morphological characters of unbranched acellular stalks and spherical spores. Although *A. ellipticum *(Group 2B) also has a simple acellular stalk, its spores are elliptical.

In contrast, Group 2B is the most heterogenous of all the major groups, more so in the new phylogeny with four additional species of Dictyostelids. With the additional species and strong internal resolution, this is one group in which it is now possible to reconstruct some of the major events in its evolution. The early branching position of *A. ellipticum *and its strong similarity to the Group 2A Acytostelids suggests that the last common ancestor of Group 2B had an acytostelid morphology. That is, it was probably a small delicate species with a non-cellular sorocarp stalk. Since all other Group 2B species have sorocarps with cellular stalks and lateral branches, these traits must have evolved soon after the *A. ellipticum *lineage split off. This irregularly branched Dictyostelids morphology then existed for some time, during which the *D. gloeosporum *and *D. boreale *lineages split off, eventually giving rise to the regularly spaced symmetrical whorls of the Polysphondylids. This polysphondylid morphology appears to have diversified rapidly. However, there is evidence of only a single reversion of the polysphondylid morphology to irregular branching, in a common ancestor to the *D. gloeosporum*, *D. granulosum, D. rotatum*, *D. flexuosum *clade of Group 2B1 (1.00 biPP, 100% mlBP, Figure 2B).

Group 2B species also show a trend toward having unconsolidated granules in their spores and of having a filose sorophore tip (Additional file 1). However in some cases the polar granules are consolidated polar and have different refractivity (e.g., *D. oculare*), or they may be distributed across the spore (e.g. *D. flexuosum*), or, in the case of *D. gloeosporum*, they can be irregular or sometimes lacking altogether. Group 2B could possibly also share the use of glorin as an acrasin, although acrasin identity is so far known only for the Polysphondylids [28,29]. More chemical studies would be needed to pin down these groups.

### Group 3

Group 3 is the most species poor of the original four major groups, and only four new species are added here (Figure 2C). Nonetheless, the species in this group vary widely in size and sorocarp morphology, ranging from the tiny *D. minutum *to the larger and structurally relatively complex crampon-based species. One common feature of the Group is the presence of consolidated polar granules inside the spores, which is found in all species except *D. minutum*. In addition, none of the sporophores in this group are as robust as most of the sporophores in Group 4. The crampon-based species of Group 3 with digitated basal structures to support their sorocarps, form the molecular very distinct subclade 3B (1.00 biPP, 100% mlBP, Figure 2C) [17]. The length of the branch leading to subclade 3B suggests that intermediate branches in the evolution of this relatively complex morphology might yet be found.

### Group 4

Group 4 is the most speciose and intensively studied major group of Dictyostelia, especially the model organism *D. discoideum*. The group was previously characterized for three main common characters: 1) large sorocarps; 2) solitary and unbranched fruiting bodies; 3) spores that lack polar granules [11]. However, some of the new species added to the group include exceptions to all three traits. In terms of size, the group now includes *D. valdivianum*, one of the smaller described species of dictyostelids. In terms of sorocarp morphology, *D. austroandinum *and *D. valdivianum *have clustered and coremiform sorocarps, respectively, and *D. ammophilum *with a characteristic pattern of irregular lateral branches formed by "blebbing" of the myxamoebae (Additional file 1) [30]. The latter pattern of branching is similar to that of the Group 1 species, *D. aureostipes*. Finally three new Group 4 species, *D. ammophilum, D. austroandinum*, and *D. valdivianum *not only have polar granules in their spores, but *D. ammophilum *spores have both consolidated and unconsolidated granules [30,20].

### Three complexes

The polycephalum and polycarpum "complexes" have very few taxa, and as a result they are morphologically extremely homogenous. However, they both also appear to represent very deep and ancient lineages so there are probably many additional species to be found. The "*polycarpum*" complex is especially intriguing as it appears to be a very ancient lineage, being sister group to a large portion of the tree (Figure 1). These species have clustered sorocarps that adhere near the base, multiple sorogens arising from a single aggregation and polar granules. The "*polycephalum*" complex also appears to be fairly ancient and is now strongly supported as the sister group to Groups 3+4 and the "*violaceum*" complex (0.95 biPP, 99% mlBP, Figure 1). This taxon is characterized by small coremiform fruiting bodies, very long thin slugs and spore granules that are sometimes polar.

The "*violaceum *complex" on the other hand is very morphologically diverse, including as it does both Polysphondyliums and a Dictyostelid (Figure 1). Nonetheless, these species have two strong common traits, which are violet or purple-pigmented sorocarps and consolidated polar granules inside their spores. While the one Dictyostelid in this group, *D. laterosorum*, lacks the whorled branching pattern of the polysphondylids, it does have multiple sori that are sessile along the stalk, which is similar to *D. rosarium *in Group 4C5. The initial development of the fruiting body is similar in both *P. violaceum *and *D. laterosoum*. However, in the polysphondylids there is a subsequent subdivision of labor in the lateral whorls, with both spore and stalk cells developing, while in *D. laterosoum *(and *D. rosarium*) all laterally distributed amoebae become only spore cells [31,32].

### The root

There are currently two proposed positions for the root of Dictyostelia, either placing Group 1 as the earliest diverging lineage (root 1, Figure 1, [11]) or splitting the taxon roughly equally along the branch connecting Groups 1 + 2 and Groups 3 + 4 (root 2). Analyses of SSU rDNA and alpha-tubulin give either alternative without definitive statistical support and depending on the outgroup used [11]. Although phylogenetic analysis of mitochondrial genes showed strong support for root 2, this analysis included only four taxa, an extremely distant outgroup and a large proportion of neutral codon positions that are likely to be saturated at this evolutionary depth (>400 myr) [33]. Figure 1 is rooted consistent with the initial phylogeny [11] and is used here simply to maintain continuity until a more robust answer can be found. This will probably require multiple gene sequences from a broad taxonomic sampling of both ingroup and outgroup taxa.

## Conclusions

The initial Dictyostelia phylogeny showed many long unbroken branches and large distances between the major lineages. Four years later, with nearly double the number of species in the tree, many of the long branches have been broken and additional major lineages are apparent (Figure 1). This suggests that a large diversity of species remain to be discovered. While many of these may exhibit new unique morphologies, many others may be morphologically indistinguishable from currently known species. Nonetheless these species can be molecularly quite distinct, and probably differ in many traits that are not associated with morphology or not apparent at the level of morphology currently examined.

The 50 new species added here show that the few patterns tentatively identified at that time are mostly invalidated. There is also a high level of cryptic species found throughout the taxon, with the majority of species still not examined for multiple isolates. Nonetheless all the new species reported here were initially identified based on morphology alone, and their uniqueness is confirmed by phylogeny. In addition, morphological consistencies can be found at finer levels. Prominent examples of this are species with crampon-based sporophores in Group 3 (clade 3B, Figure 2C), the "gigantic" species in Group 4 (clade 4A, Figure 2D), robust pigmented species with side branches in the *violaceum *complex (Figure 1), species with clustered fruiting bodies in group 1, species with twisted stalks (clade 4C3, Figure 2D), the pale polysphondylids (clade 2B, Figure 2B), and most of the acytostelids (clade 2A, Figure 2B) (Additional file 1).

Since morphological patterns can be identified for more limited subgroups, morphological evolution seems to be at least moderately conservative in Dictyostelia. The lack of deeper morphological patterns should perhaps not be entirely unexpected given the small numbers of characters, the essentially simple nature of many of them, and the apparent antiquity of the group [24]. Nonetheless, the future discovery of additional species, together with extensive genome sequencing, should lead to a better understanding of the mechanisms and evolutionary forces shaping them.

## Methods

Samples were collected primarily from the soil/humus zone at the surface of the ground, plus a few samples from "canopy soil" [*sensu *34] often found at the bases of epiphytes in moist temperate and tropical forests. All samples were stored in sterile containers for transport. In the laboratory, samples were processed following standard procedures [35]. In brief, sample material was dispersed in sterile distilled water (1/50 dilution factor), and small aliquots (0.5 ml) plated on hay infusion agar [1], with a bacterial suspension (0.4 ml *Escherichia coli *or *Klebsiella aerogenes*). Soil suspensions prepared in this manner contain viable dictyostelid propagules in the form of spores, active amoebae or encysted resting amoebae (microcysts), and all of which can yield viable fruiting colonies [1]. Isolates of potential interest were subcultured from spores obtained directly from newly formed fruiting bodies. All types have been deposited in the Dicty Stock Center Home (http://www.dictybase.com).

### Cell culture and genomic DNA extraction

DNA was extracted from colonies grown on SM plates (Standard Medium - 20 g/L peptone; 2 g/L yeast extract; 20 g/L glucose; 2 g/L MgSO4; 3.8 g/L KH2PO4; 1.2 g/L K2HPO4; 2% agar). Cells were collected from the edges of plaques with a sterile tip, mixed with DNA Extraction solution (Epicentre) and heated 30 min at 60°C, followed by 8 min at 98°C. Cell lysates were used directly for PCR amplification.

### PCR amplification and DNA sequencing

A ~2000 base pair (bp) fragment of SSU rDNA was amplified using the same primers as in [11]. The PCR program consisted of 5 minutes at 95°C, followed by 30 cycles of 30 seconds at 95°C, 1 minute at 56°C, and 2 minutes at 72°C, with a final elongation step of 10 minutes at 72°C. Following amplification, PCR products were separated on 1% agarose gels and DNA purified with MultiScreenHTS Vacuum Manifold and MultiScreen-PCR96 Filter Plate from Millipore (http://www.millipore.com). The extracted DNA was then directly sequenced using the same primers used for amplification by Macrogen (Korea) on an ABI 373 sequencer. Sequences were edited with the program Sequencher version 3.0 (Gene Codes Corporation Inc.). Edited sequences were manually added to an existing alignment [17]. Only unambiguously aligned regions were used for phylogenetic analysis.

### Phylogenetic analysis

Phylogenetic analyses were carried out using Bayesian inference with MrBayes version 3.1.2 [36] and maximum likelihood using RaxML version 7.0.4 [37,38]. MrBayes analyses utilized the MC3 search algorithm and the GTR+I+G model [17], with parameters determined through the course of the run. Searches were conducted with two independent sets of four chains for 1-10 million generations, with results saved every 10 generations. At the end of the run (split frequency less than 0.01), a 20% burnin was discarded before determining the optimal topology and posterior probabilities (biPP) of clades. Maximum likelihood analyses consisted of 1000 bootstrap replicates (mlBP) using the GTR+I+G model with parameters determined from a BioNJ starting tree.

## Authors' contributions

MR and SLB designed the study. MR, SLS, JCL and JCC collected the samples. MR, JCC and JCL studied the morphological traits. MR did the molecular lab work and performed the phylogenetic analyses. MR and SLB drafted the manuscript. JCC, JCL and SLS revised the manuscript. All authors approved the final manuscript.

## Supplementary Material

Additional file 1 **Information about the new species and isolates included in this phylogeny**. Morphological traits, geographical origins, GeneBank acession numbers and references for new species and isolates included in this phylogeny [39-43]. Click here for file
