# Supplementary material for: An expanded phylogeny of social amoebas (Dictyostelia) shows increasing diversity and new morphological patterns
Source: BMC Evol Biol. 2011 Mar 31;11:84. doi: 10.1186/1471-2148-11-84 (PMC3073913; doi:10.1186/1471-2148-11-84)
Supplement: Additional file 1 — Information about the new species and isolates included in this phylogeny. Morphological traits, geographical origins, GeneBank acession numbers and references for new species and isolates included in this phylogeny [39-43]. [file 1471-2148-11-84-S1.DOC]

| Group | Species name | Isolate | ATCC No. | Dicty Stock Center No. | Publication | Origin | GeneBank acession number | Sorocarp habit | Spore size (µm) | Polar granules | Branching pattern |
| --- | --- | --- | --- | --- | --- | --- | --- | --- | --- | --- | --- |
| 1 | D. myxobasis | NT2A | MYA-3817 | DBS0238801 | (Landolt et al, 2008) | Australia | HQ141522 | solitary to clustered | 6—9 x 2.5--4 | consolidated | occasionally branched |
| 1 | D. macrocarpum | MGE2 | - | DBS0235722 | (Vadell and Cavender, 2007) | Argentina | HQ141519 | solitary to tightly clustered | 4–6 x 2–3 | consolidated polar to subpolar | irregularly branched |
| 1 | D. amphisporum | BM9A | MYA-3273 | DBS0235721 | (Cavender et al, 2005) | USA | HQ141521 | loosely clustered | larger: 4.8–6.3 x 2.8–3.5; smaller 3–4.5 x 1.5–2.8 | consolidated | mostly unbranched |
| 1 | D. fasciculoideum | Cavender Puelo 2 | MYA-3806 | DBS0238804 | (Vadell et al, 2011) | Argentina | GQ496157 | tightly clustered to solitary | 5–6 x 2.5–3 | consolidated | unbranched or with 0.7–3 mm long branches |
| 1 | D. boomerasporum | K26B | MYA-3802 | - | (Landolt et al, 2008) | Australia | HQ141520 | clustered | 4-9.5 x 2-3.5 | consolidated | irregularly spaced branches |
| 1 | D. THC11X | THC11X | - | - | - | Thailand | HQ141523 | solitary | 8.15 x 4.26 | consolidated | generally unbranched |
| 1 | D. TH1A | TH1A | - | - | - | Thailand | HQ141515 | solitary | 7.07 x 3.29 | consolidated | generally unbranched |
| 1 | D. TH18B | TH18B | - | - | - | Thailand | HQ141517 | solitary | 7.76 x 3.77 | consolidated | generally unbranched |
| 1 | D. TH39A | TH39A | - | - | - | Thailand | HQ141518 | clustered | 7.12 x 3.56 | consolidated | irregular branching |
| 1 | D. TAS30A | TAS30A | - | DBS0267156 | - | Tasmania | HQ141516 | clustered to solitary | 6-8 x 3-4 | consolidated | irregular branching |
| 2B | D. flexuosum | AU4B | MYA-3807 | - | (Landolt et al, 2008) | Australia | HQ141500 | clustered to solitary | 3.5-5 x 3-4 | Unconsolidated, spaced throughout | unbranched |
| 2B | D. rotatum | QC2C | MYA-3820 | - | (Landolt et al, 2008) | Australia | HQ141501 | solitary to clustered | 4.5-7x 2.7-4 (5.28x3.4) | unconsolidated | generally with long branches |
| 2B | D. granulosum | MF5A | MYA-3809 | - | (Landolt et al, 2008) | Australia | HQ141502 | loosely clustered | 5-6.5 x 3-4 (5.34 x 3.50) | Unconsolidated, sometimes spaced throughout, but mostly at poles. |  |
| 2B | P. laos 3 | laos 3 | - | - | - | Laos | HQ141496 | mostly clustered | 3-5 x 6-8 | unconsolidated | whorls |
| 2B | D. boreale | BSB10A | - | - | (Romeralo et al, 2010a) | Alaska | HQ141499 | clustered, or solitary (centripetal growth tendency) | 5.3-9.2 x 2.1-3.2 (7.0 x 2.6) | unconsolidated | unbranched |
| 2B | D. boreale | BSB10B | MYA-4278 | - | (Romeralo et al, 2010a) | Alaska | FJ940744 | clustered, or solitary (centripetal growth tendency) | 5.3-9.2 x 2.1-3.2 (7.0 x 2.6) | unconsolidated | unbranched |
| 2B | P. TH12A | TH12A | - | - | - | Thailand | HQ141504 | solitary | 10.52 x 4.90 | unconsolidated | short with usually a singler whorl |
| 2B | P. multicystogenum | AS2 | - | - | (Kawakami and Hagiwara, 2008) | West Africa | HQ141506 | solitary or clustered | 5.0-6.0 x 2.9-3.5 | unconsolidated | 1–7(–11) nodes |
| 2B | P. australicum | NB1AP | MYA-3833 [as Polysphondylium luteo-stipes] | DBS0238799 | (Landolt et al, 2008) | Australia | HQ141508 | solitary to clustered | 7.5-9 x 4-4.5 (8.54 4.28 ) | unconsolidated | 2 or 3 whorls |
| 2B | P. candidum | Landolt bsb6b | - | - | another isolate from that species | USA | HQ141498 | solitary to clustered | 8-11.4 x 3.7-5.5 | unconsolidated | 1-6 whorls |
| 2B | P. asymmetricum | Landolt HN20C | - | - | another isolate from that species | Honduras | HQ141503 | clustered or coremiform | 6.8 x 3.9 | unconsolidated and consolidated | 5-10 whorls |
| 2B | P. colligatum | Landolt HN13C1 | - | - | another isolate from that species | Honduras | HQ141505 | tightly clustered, coremiform | 5-7 x 3-4 (5.5x3.6) | unconsolidated | 4-25 whorls |
| 2B | P. Tikaliense | Landolt HN1C1 | - | - | another isolate from that species | Honduras | HQ141509 | - | - | - | - |
| 2B | P. stolonicoideum | K12A | MYA-3835 | DBS0238800 | (Landolt et al, 2008) | Australia | HQ141507 | solitary to clustered | 5.5-6.5 x 3-4 | unconsolidated polar, irregularly spaced | 2--7 whorls |
| 2B | D. oculare | landolt | - | - | (Cavender et al, 2005) | USA | HQ141497 | - | - | - | - |
| 2A | A. singulare | FDIB | MYA-3272 | - | (Cavender et al, 2005) | USA | HQ141514 | solitary | 4–5.5 | few peripheral granules | generally branched |
| 2A | A. magnisorum | 08A | MYA-3270 | - | (Cavender et al, 2005) | USA | HQ141513 | clustered | 3.5–5, mostly 4–4.5 | some peripheral granules | unbranched |
| 2A | A. amazonicum | HN1B1 | - | - | (Cavender et al, 2005) | Honduras | HQ141511 | solitary to clustered | 4 x 7 | vesicles | branched |
| 2A | A. amazonicum | landolt X | - | - | (Cavender et al, 2005) | Honduras | HQ141510 | solitary to clustered | 4 x 7 | vesicles | branched |
| 2A | A. leptosomum | 212rjb | - | - | (Romeralo et al, 2007) | Portugal | HQ141512 | clustered | 5 x 7 | none | unbranched |
| 3 | D. ibericum | 214rjb | - | DBS0304360 | (Romeralo et al, 2009) | Portugal | HQ141495 | generally gregarious, sometimes solitary and clustered | 6–7 x 3.5–4 (median: 6.2 x 4 ) | consolidated spaced throughout; unconsolidated also present | unbranched |
| 3 | D. Ohioense | Okla4C | - | DBS0238805 | (Cavender and Vadell, 2006) | USA | HQ141493 | solitary to clustered | 4.5-5.5 x 3-3.5 | consolidated | unbranched |
| 3 | D. radiculatum | ML5A | MYA-4248 | DBS0238798 | (Landolt et al, 2008) | Australia | HQ141494 | solitary to clustered | 5—11x 2.5--4.5 (median: 6.78 x4.29) | mostly with large consolidated polar; some with spaced granules throughout | generally unbranched |
| 3 | D. TH14B | TH14B | - | - | - | Thailand | HQ141491 | solitary | 6.88 x 3.34 | consolidated | generally unbranched |
| 3 | D. TH8C | TH8C | - | - | - | Thailand | HQ141492 | clustered | 7.25 x 3.61 | unconsolidated | monochasioides-like branching |
| 4 | D. austroandinum | Cavender Blest 5 | MYA-3825 | - | (Vadell et al, 2011) | Argentina | GQ496158 | generally solitary, sometimes tightly clustered in groups of 2 to 3, then coremiform | 5–8 x 2.5–4.5 (6.32 x 3.40 ) | numerous small scattered granules | none or 1–4 distantly spaced branches |
| 4 | D. chordatum | Cavender Moreno 7 | MYA-3810 | DBS0238803 | (Vadell et al, 2011) | Argentina | GQ496159 | solitary | –9(–10) x 4–5.5(–6) ( 8.54 x 4.64) | dispersed granules | unbranched to branched, with large branches near the base and smaller ones in the upper portion |
| 4 | D. leptosomum | NZN49A | - | DBS0266749 | (Cavender et al, 2002) | New Zealand | HQ141480 | solitary to clustered, with a stoloniferous habit | 5–6.5 × 2–3, mostly 5.5 × 3.0 | vesicles | unbranched or irregularly branched |
| 4 | D. ammophilum | KBK4A | - | - | (Romeralo et al, 2010a) | Alaska | HQ141478 | solitary | 5.0-7.6 x 2.6-3.9 | consolidated and unconsolidated polar(occasionally sub-polar) | irregular lateral branches by “blebbing” of myxamoebae |
| 4 | D. ammophilum | NW2B | - | - | (Romeralo et al, 2010a) | Alaska | FJ940745 | solitary | 5.0-7.6 x 2.6-3.9 | consolidated and unconsolidated polar(occasionally sub-polar) | irregular lateral branches by “blebbing” of myxamoebae |
| 4 | D. valdivianum | Cavender Vald 3C | MYA-3823 | - | (Vadell et al, 2011) | Argentina | GQ496155 | generally clustered | 4–6(–7) x 2.5–3(–3.5) (5.15 x 2.98) | small to medium polar granules | unbranched or with 1 to many short branches |
| 4 | D. quercibrachium | NZ201B | - | DBS0266754 | (Cavender et al, 2002) | New Zealand | HQ141479 | solitary to clustered | 4.0–7.5 × 2–4 | vesicles | unbranched to multi-branched |
| 4 | D. laos 5 | laos 5 | - | - | - | Laos | HQ141484 | mostly solitary | 3-4 x 6-8 | granules | unbranched |
| 4 | D. laos 1 | laos 1 | - | - | - | Laos | HQ141483 | solitary, clustered, gregarious. | 3-4 x 7-9 | granules | branched |
| 4 | D. gargantuum | Cavender Puelo 1 | MYA-3808 | - | (Vadell et al, 2011) | Argentina | GQ496161 | solitary, gigantic | 6–11.5(–12) x 3–4(–4.5) (8.58 x 3.80) | scattered granules | unbranched but sometimes with delicate broken branches |
| 4 | D. mucoroides | Sweden20 | - | - | - | Sweden | HQ141482 | solitary | - | none | unbranched |
| 4 | D. purpureum | cavender | - | - | - | USA | HQ141481 | solitary | - | none | unbranched |
| polycephalum complex | D. polycephalum | Landolt #2132 B-9c | - | - | another isolate from that species | USA | HQ141489 | clustered to coremiform | 6-7.5 x 3-3.5 | consolidated | branched |
| polycephalum complex | D. polycephalum | Landolt #1675 GUAM | - | - | another isolate from that species | Guam | HQ141490 | clustered to coremiform | 6-7.5 x 3-3.5 | consolidated | branched |
| polycephalum complex | D. polycephalum | Landolt #1130 SS3B | - | - | another isolate from that species | Bahamas | HQ141488 | clustered to coremiform | 6-7.5 x 3-3.5 | consolidated | branched |
| violaceum complex | P. patagonicum | Cavender H-H 1 | MYA-3834 | DBS0238806 | (vadell et al, 2011) | Argentina | GQ496156 | solitary to clustered, frequently coremiform stoloniferous habit | 6–7.5 x 3.3–4 | consolidated | 1-5 whorls. long branches when prostrate |
| violaceum complex | P. laos 4 | laos 4 | - | - | - | Laos | HQ141485 | mostly solitary | 3-3.5 x 6-7.5 | unconsolidated | whorls |
| violaceum complex | P. tibet | 10A | - | - | - | Tibet | HQ141487 | mostly gregarious | 2.9-4.2 x 5.8-8.9 | unconsolidated | whorls |
| violaceum complex | P. violaceum | 209 | - | DBS0236814 | another isolate from that species | USA | HQ141486 | solitary to clustered | 5-7 x 2.5-3 | consolidated | 3-8 whorls |
